# Plasma Aldosterone Concentration as a Determinant for Statin Use among Middle-Aged Hypertensive Patients for Atherosclerotic Cardiovascular Disease

**DOI:** 10.3390/jcm7110382

**Published:** 2018-10-24

**Authors:** Jui-Hsiang Lin, Yu-Feng Lin, Wei-Jie Wang, Yuh-Feng Lin, Shih-Chieh Jeff Chueh, Vin-Cent Wu, Tzong-Shinn Chu, Kwan-Dun Wu, on behalf of the Taiwan Primary Aldosteronism Investigation (TAIPAI) Study Group

**Affiliations:** 1 Graduate Institute of Clinical Medicine, College of Medicine, Taipei Medical University, Taipei 110, Taiwan; terencelin78@gmail.com (J.-H.L.); linyf@shh.org.tw (Y.-F.L.); 2 Division of Nephrology and Sinwu Branch, Department of Internal Medicine, Taoyuan General Hospital, Ministry of Health and Welfare, Taoyuan 330, Taiwan; mrwwj.tw@yahoo.com.tw; 3 Faculty of Medicine, National Yang-Ming University, Taipei 112, Taiwan; 4 Graduate Institute of Clinical Medicine, National Taiwan University College of Medicine, Taipei 100, Taiwan; dr.yufenglin@gmail.com; 5 Department of Internal Medicine, Division of Nephrology, National Taiwan University Hospital, Taipei 100, Taiwan; tschu@ntu.edu.tw (T.-S.C.); kdwu@ntuh.gov.tw (K.-D.W.); 6 Division of Hospital Medicine, Department of Internal Medicine, National Taiwan University Hospital, Taipei 100, Taiwan; 7 Department of Biomedical Engineering, Chung Yuan Christian University, Taoyuan 320, Taiwan; 8 Ministry of Health and Welfare, Shuang Ho Hospital, Taipei Medical University, Taipei 235, Taiwan; 9 Cleveland Clinic Lerner College of Medicine and Glickman Urological and Kidney Institute, Cleveland Clinic, OH 441, USA; jeffchueh@gmail.com

**Keywords:** plasma aldosterone concentration, atherosclerotic cardiovascular disease, ASCVD, statin, primary hyperaldosteronism, essential hypertension

## Abstract

The use of statin therapy on the prevention of atherosclerotic cardiovascular disease (ASCVD) is recommended by the American College of Cardiology (ACC) and the American Heart Association (AHA); nevertheless, its validation on primary aldosteronism (PA) patients has not been reported. We investigated the risk of incident ASCVD in middle-aged patients with PA compared with essential hypertension (EH) based on ACC/AHA recommendations. We enrolled 461 PA patients and 553 EH patients. Even though the ratio of metabolic syndrome in each group was similar, the PA group had higher systolic blood pressures, higher low-density lipoprotein levels, higher plasma aldosterone concentration (PAC), lower high-density lipoprotein levels, and higher 10-year ASCVD compared to the EH group. The discriminative power for predicting ASCVD by the recommended statin use from the ACC/AHA guidelines was proper in the PA group (i.e., under the receiver operating characteristic curve (95% confidence interval; 0.94 (0.91–0.96)). The generalized additive model showed patients with PAC higher than 60 ng/dL accompanying the standard timing of the statin use suggested by the ACC/AHA. The ACC/AHA guidelines have good discriminative power in the prediction of middle-aged high-risk hypertensive patients, while PAC identifies those high-risk individuals who may benefit from early statin therapy.

## 1. Introduction

Primary aldosteronism (PA), characterized by the autonomous production of aldosterone, is a common and potentially curable disease of secondary hypertension [1,2]. PA leads to 5−13% of patients developing resistant hypertension [3]. On the other hand, aldosterone increases oxidative stress and inflammation, contributing to impaired pancreatic beta-cell function and diminished skeletal muscle insulin metabolic signaling. PA patients have a high incidence of cardiovascular (CV) events in comparison with essential hypertension (EH) patients [4,5]. Furthermore, the higher prevalence of metabolic syndrome was expected as a result of the increased rate of CV events observed in patients with PA compared to EH [4,6].

The American College of Cardiology (ACC) and the American Heart Association (AHA) released new guidelines for statin therapy based on the latest cholesterol management guidelines [7]. The ACC/AHA guidelines introduced a prediction model and lowered the threshold for treatment with statins to a 10-year atherosclerotic cardiovascular disease (ASCVD) risk of 7.5%. The ACC/AHA guidelines for the management of cholesterol increase the number of adults who would be eligible for statin therapy [8] and have better predictive value for cardiovascular (CV) events [9] compared with the previous Adult Treatment Panel III (ATP-III) guidelines [2]. Based on a Cardiovascular Life Expectancy Model to estimate the advantages of risk factor modification in the prevention of CV events, forecasting advantages of using statin for the therapy of hyperlipidemia have shown that middle-aged patients have the highest level of benefits [10]. However, the implications of this updated guideline for statin recommendations have not been addressed in a population at high risk for a CV event, mainly primary aldosteronism patients.

Middle-aged hypertensive patients with PA and EH were prospectively studied at diagnosis. This study aimed to evaluate and validate the incident ASCVD risk, defined as the development of a CV and stroke event within a 10-year period, taking into account several risk factors simultaneously, according to the ACC/AHA guidelines, in patients with PA and EH. Furthermore, we studied the relationship of plasma aldosterone concentration (PAC) and the use of statins based on the suggestion of the guidelines for the prevention of ASCVD.

## 2. Materials and Methods

### 2.1. Study Design and Participants

A total of 1197 patients with hypertension who underwent confirmatory tests were recorded in the Taiwan Primary Aldosteronism Investigation (TAIPAI) database between January 2007 and March 2013 [11,12,13]. The database was constructed for quality assurance at two medical centers and their four affiliated hospitals in different cities in Taiwan [14]. Some cautions should be considered prior to depicting the paired PAC and plasma renin activity values. While patients with hypertension have outpatient clinic follow-up, the physicians should hold certain antihypertensive drugs including a direct renin inhibitor such as aliskiren, β-adrenergic blockers such as labetalol, central α-2 agonists such as clonidine and methyldopa, and dihydropyridine calcium-channel blockers [15]. All antihypertensive medications were discontinued for at least 21 days before screening tests. Doxazosin and/or diltiazem were administered to control markedly high blood pressure when required [15]. All patients included in the statistical analysis were statin-naive patients at the time of inclusion. The biochemistry data were obtained after the drug-holding period before PA treatment. Patients with an abnormal aldosterone-renin ratio (ARR) were confirmed to have PA with a saline infusion test, and subsequent imaging studies were performed for subtype identification. PA confirmation and subtype studies were established in hypertensive patients according to the standard protocol of TAIPAI, including adrenal venous sampling and 131I-iodocholesterol scintigraphy with single-photon emission computed tomography/ computed tomography imaging [15,16,17,18,19,20]. Patients diagnosed with EH were enrolled as controls [16].

**Figure 1:**
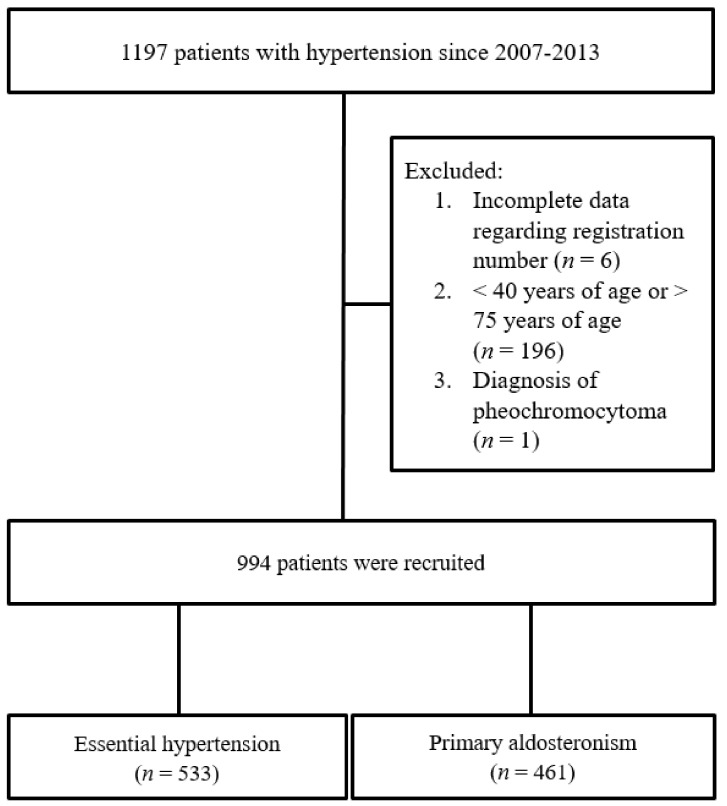
Flow diagram of the study groups.

Our study used a validated algorithm to identify 1197 patients with hypertension, and further enrolled patients with hypertension aged between 40 and 75 (Figure S1). Two hundred and three patients were excluded for the following reasons: incomplete lipid profile or lipid-lowering therapy (*n* = 6); <40 years of age or >75 years of age (*n* = 196); and diagnosis of pheochromocytoma (*n* = 1). An observational study was conducted involving 994 patients (473 men and 521 women; age range, 40–75 years) with detailed comorbidities and lipid profiles (Figure 1). The following covariates at the time of study entry were considered as potential confounders: age, sex, diabetes mellitus (DM), body mass index (BMI), smoking status, and estimated glomerular filtration rate using the Chronic Kidney Disease Epidemiology Collaboration (CKD-EPI) equation. We evaluated left ventricular hypertrophy in accordance with left ventricular mass index (LVMI) and relative wall thickness (RWT). The cut point value of increased RWT derived from the upper limits of the normal population is 0.45. The reference cut off limits of LVMI consider that men are less than or equal to 131 g/m^2^ and women are less than or equal to 100 g/m^2^ [21]. Based on the Health Promotion Administration, metabolic syndrome is defined as the presence of three or more of the following components: (i) waist circumference is greater than 90 cm for males and 80 cm for females, (ii) systolic blood pressure is greater than 130 mmHg or diastolic blood pressure is greater than 85 mmHg, (iii) fasting blood glucose levels are greater than 100 mg/dL, (iv) triglyceride levels are greater than 150 mg/dL, (v) high-density lipoprotein cholesterol levels are less than 40 mg/dL for males or 50 mg/dL for females [22]. We validated the 10-year ASCVD by the ACC/AHA guidelines (Figure S1).

### 2.2. Outcome Measures

The primary outcome was an incident ASCVD defined as a composite of incident myocardial infarction, other coronary heart disease, or stroke based on the ACC/AHA guidelines.

### 2.3. Ethics Committee Approval

The Institutional Review Board of the National Taiwan University Hospital (Taipei, Taiwan; No. 200611031R) approved this study. All participants signed a written informed consent form before inclusion in the study.

### 2.4. Statistical Analysis

Data are expressed as a mean ± standard deviation (SD). Qualitative criteria were compared using Fisher’s exact test. Continuous data were analyzed with the two-tailed Student *t*-test. We estimated the total number of persons in the two groups (EH and PA) who would be eligible for statin therapy on the basis of the ACC/AHA guidelines, further stratifying the persons according to the recommendation for statin therapy. To evaluate the effect of PAC on the risk of recommended statin use, we further adopted a generalized additive model (GAM) with adjustment for baseline comorbidities [18,23]. This method grants adjustments for possible non-linear effects from continuous variables. The result was shown as a function curve with values of the log odds ratio and was centered to have an average of zero over the range of the data. All analyses were performed by the R software (version 3.5.1; Free Software Foundation, Inc., Boston, MA, USA). A two-sided *p*-value < 0.05 was considered significant.

## 3. Results

### 3.1. Patient Characteristics

The characteristics of the participants with EH and PA are shown in Table 1. Both groups were matched for age, gender and BMI in the study. The average age was 57 years, and 44.9% were male. The PA group consisted of 335 adults with aldosterone-producing adenoma (APA) and 126 adults with idiopathic bilateral adrenal hyperplasia. The PA patients had higher PAC (48.2 ± 32.7 vs. 36.9 ± 83.8 ng/dL, 1.3 ± 0.9 vs. 1.0 ± 2.3 nmol/L, *p* = 0.008), higher log ARR (4.9 ± 2.0 vs. 2.9 ± 2.0, *p* < 0.001), lower renin levels (1.2 ± 4.3 vs. 4.5 ± 1.2 ng/mL/h; 28.4 ± 101.9 vs. 106.7 ± 28.4 pmol/L/h, *p* < 0.001) and potassium levels (3.7 ± 0.7 vs. 4.3 ± 2.0 mmol/L; 3.7 ± 0.7 vs. 4.3 ± 2.0 mEq/L, *p* < 0.001) than EH patients. The PA patients also had higher systolic blood pressures (151.5 ± 22.4 vs. 144.5 ± 21.5 mmHg, *p* < 0.001), higher low-density lipoprotein-cholesterol (LDL-c) levels (119.0 ± 31.9 vs. 114.6 ± 32.6 mg/dL, 3.1 ± 0.8 vs. 3.0 ± 0.8 mmol/L, *p* = 0.033), and lower high-density lipoprotein-cholesterol (HDL-c) levels (46.1 ± 13.6 vs. 48.0 ± 12.4 mg/dL, 1.2 ± 0.4 vs. 1.2 ± 0.3 mmol/L, *p* = 0.018) compared to EH patients. The proportions of patients who received aspirin therapy and were associated with DM, obesity, metabolic syndrome, and BMI were similar in both groups. The primary outcome of 10-year ASCVD was higher in PA patients than in EH patients (20.6% vs. 13.8%, *p* < 0.001) (Table 1).

### 3.2. The Predictive Power of the ACC/AHA Guidelines

Even though the estimated 10-year ASCVD risk had better discriminative power in PA patients compared to EH patients (area under receiver operating characteristic curve (AUROC) = 0.76 (0.70–0.82) vs. 0.64 (0.58–0.71)), the discriminative ability of the recommended statin use by the ACC/AHA guidelines could well predict 10-year ASCVD in both PA and EH patients (AUROC = 0.94 (0.91–0.96) vs. 0.93 (0.91–0.95)) (Table 2). Besides, the discriminative cut-off values of 10-year ASCVD risk in both PA and EH patients were all 7.5% (Table S1), which was also compatible with the published ACC/AHA guidelines on the definition of high-risk patients.

### 3.3. Effect of Plasma Aldosterone Concentration on Recommended Statin Use

**Figure 2:**
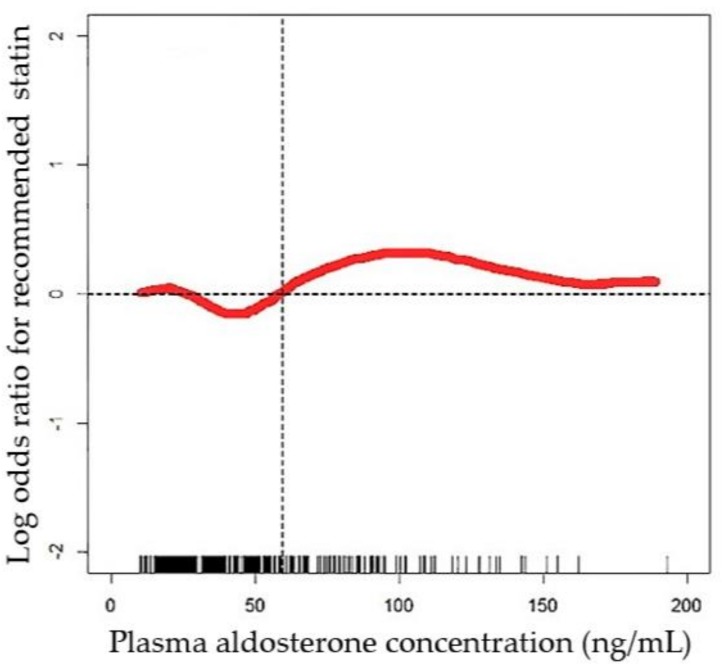
Generalized additive model (GAM) plot showing the recommendations for statin use by the 2013 ACC/AHA guidelines against plasma aldosterone concentration, incorporating the subject-specific (longitudinal) random effects. The probability of statin treatment was constructed with aldosterone level and was centered to have an average of zero over the range of the data as constructed with the GAM. Abbreviations: GAM, generalized additive model.

We evaluated the PAC in middle-aged high-risk hypertensive patients and the recommended statin use by the ACC/AHA guidelines utilizing a GAM analysis. The function curve was nonlinear and there was a significant trend approaching the recommended statin use, especially for a PAC > 60 ng/dL (Figure 2).

### 3.4. The Comparison of Middle-Aged High-Risk Hypertensive Patients by the Plasma Aldosterone Concentration

The PAC under 60 ng/dL group consisted of 848 (85.3%) adults. The PAC greater than 60 ng/dL group had higher proportions of left ventricular hypertrophy (31.2% vs. 13.2%, *p* = 0.015), lower potassium levels (3.5 ± 0.8 vs. 3.9 ± 0.7 mmol/L, 3.5 ± 0.8 vs. 3.9 ± 0.7 mEq/L, *p* = 0.016), lower fasting blood glucose levels (95.6 ± 18.2 vs. 99.9 ± 22.3 mg/dL, 5.3 ± 1.0 vs. 5.5 ± 1.2 mmol/L, *p* = 0.07), lower levels of triglycerides (129.3 ± 79.1 vs. 147.2 ± 108.3 mg/dL, 1.5 ± 0.9 vs. 1.7 ± 1.2 mmol/L, *p* < 0.001), and lower levels of estimated glomerular filtration rate (78.5 ± 31.1 vs. 80.9 ± 23.7 mL/s per 1.73 m2, *p* < 0.001) than the PA under 60 ng/dL group (Table S2).

## 4. Discussion

Our study indicated that the estimated ASCVD risk and the recommended statin use according to the algorithm by the ACC/AHA had good discriminative power in the ratio of 10-year ASCVD in both PA and EH patients. Besides, higher levels of PAC (cut-off point: PAC = 60 ng/dL) contributed to more recommended statin therapy according to ACC/AHA guidelines.

The present research is the first documented study to validate the ACC/AHA guidelines in PA patients. Patients with PA can often be established with a higher risk of left ventricular hypertrophy, myocardial fibrosis, and diastolic dysfunction than EH patients [18]. Accumulating evidence suggests that the role of PA is considered one of the more frequent etiologies of secondary hypertension. Increased levels of aldosterone-mediated effects are present in situations including heart failure, PA and excessive salt intake. Hyperaldosteronism accompanied with klotho deficiency gain recognition from a disproportionally increased risk of cardiovascular damage and metabolic diseases [5,24]. The suppression of renin by excess aldosterone is linked with evidence of vascular injury, worsening blood pressure, and renal or cardiovascular events after target management in PA patients compared with EH patients [20]. Abnormal glucose metabolism due to insulin resistance appears to be linked to aldosterone overproduction and appears to be the major contributor to metabolic dysfunction in PA patients [25]. In agreement with our report, the distribution of each component of metabolic syndrome showed a higher frequency of ASCVD risk in PA patients than EH patients, whereas there was no difference between the two groups with respect to diabetes, abdominal obesity, BMI, and smoking status [26]. This is in agreement with the fact that aldosterone excess may lead to CV damage by mechanisms independent of a hypertensive effect [27].

The efficacy of statin therapy in reducing the risk of ASCVD has been well established [28]. Baudrand and his colleagues observed that the pleiotropic effects of statins mediated aldosterone secretion in adrenal cells. Lipophilic statins had a greater effect in suppressing aldosterone production than a hydrophilic statin. Further, potassium-stimulated aldosterone could be interrupted immediately by a 33% decrease in statins; the condition suggests that statins reduce PAC from the angiotensin II receptor type 1. Consistent with the concept, mesangial cells represent that statins inhibit acute aldosterone production induced by angiotensin II in vitro, in addition to long-term statin treatment blocking the expression of aldosterone synthase in the kidney in rats [29]. Recent evidence also demonstrates that proper statin use is related to decreased CV morbidity and mortality, particularly with high-risk patients [28,29]. This fact emphasizes the importance of better biomarkers for recommended statin therapy in high-risk patients. Our finding of PAC cut-off points (PAC > 60 ng/dL) provides the opportunity for clinicians to identify those high-risk individuals who may benefit from targeted patient counseling and early statin therapy.

Clinicians should pay attention to not only the estimated ASCVD risk, but also the presence or absence of metabolic syndrome in high-risk patients. Metabolic syndrome is a complex condition caused by insulin resistance accompanying abnormal adipose function. It consists of the risk factors for coronary heart disease, as well as for diabetes [30]. It is well known that higher prevalence of metabolic syndrome is found in PA patients, as well as the favorable effect of aldosterone excess removal on glucose metabolism alterations and left ventricular mass index [31,32]. Among the components of metabolic syndrome, lower HDL-c and hypertension were prevalent in PA patients in our study. Besides, we further provided more evidence that factors of metabolic syndrome in the ACC/AHA guidelines helps to predict 10-year ASCVD events. 

There were some limitations in our study. Even though this was a prospective study, we did the retrospective analysis of the estimated ASCVD risk according to the ACC/AHA guidelines. The lack of information on lipid values limited the recruited patients. Second, the registration of smoking was based on self-reports, and therefore there may be underestimated. Finally, we have concentrated on the population between 40 and 75 years of age because the new guidelines are unknown outside this age range. Furthermore, a new recommended statin use model based on PAC level needs more studies on indicator performance and cost-effectiveness analyses.

In conclusion, the recommended statin uses by the ACC/AHA guidelines had good discriminative power on both PA and EH patients. Besides, patients with elevated aldosterone levels (PAC > 60 ng/dL) are compatible with the recommended timing of statin use on ASCVD control in middle-aged high-risk patients. We conclude that in clinical practice, the estimated ASCVD risk by the ACC/AHA guidelines should be evaluated for both primary and secondary ASCVD prevention, especially in PA patients. Because proper statin therapy is essential for clinical benefit, promoting optimal drug utilization based on PAC in all hypertensive patients is a reasonable strategy. Further influence of statin therapy on correcting metabolic abnormalities in middle-aged high-risk patients warrants further study.

## Figures and Tables

**Table 1:** Demographic characteristics of enrollees.

	EH (*n* = 533)	PA (*n* = 461)	*p*
Age (years)	57.0 ± 9.5	57.4 ± 8.4	0.484
Male gender (%)	266 (49.9)	207 (44.9)	0.115
Body mass index (Kg/m^2^)	25.4 ± 4.0	25.4 ± 3.6	0.911
Obesity (%)	65 (12.2)	48 (10.4)	0.377
Waist circumference (cm)	83.8 ± 11.0	82.8 ± 10.9	0.742
Systolic blood pressure (mmHg)	144.5 ± 21.5	151.5 ± 22.4	<0.001 *
Diastolic blood pressure (mmHg)	86.1 ± 13.1	91.0 ± 14.1	<0.001 *
Smoking status (%)	64 (12.0)	68 (14.8)	0.204
Diabetes mellitus (*n*, %)	60 (11.3)	69 (15.0)	0.083
Left ventricular hypertrophy (*n*, %)	53 (9.9)	90 (19.5)	<0.001 *
**Laboratory data**			
PAC (ng/dL) (nmol/L)	36.9 ± 83.81.0 ± 2.3	48.2 ± 32.7 1.3 ± 0.9	0.008 *
PRA (ng/mL/h) (pmol/L/h)	4.5 ± 1.2 106.7 ± 28.4	1.2 ± 4.3 28.4 ± 101.9	<0.001 *
Log ARR	2.9 ± 2.0	4.9 ± 2.0	<0.001 *
Potassium (mmol/L) (mEq/L)	4.3 ± 2.0 4.3 ± 2.0	3.7 ± 0.7 3.7 ± 0.7	<0.001 *
Fasting blood glucose (mg/dL) (mmol/L)	99.2 ± 20.0 5.5 ± 1.1	101.9 ± 25.6 5.7 ± 1.4	0.108
Total cholesterol (mg/dL) (mmol/L)	197.0 ± 35.8 5.1 ± 0.9	194.7 ± 38.1 5.0 ± 1.0	0.328
Low density lipoprotein (mg/dL) (mmol/L)	114.6 ± 32.6 3.0 ± 0.8	119.0 ± 31.9 3.1 ± 0.8	0.033 *
Low density lipoprotein ≥ 190 mg/dL (*n*, %)	12 (2.3)	9 (2.0)	0.744
High density lipoprotein (mg/dL) (mmol/L)	48.0 ± 12.4 1.2 ± 0.3	46.1 ± 13.6 1.2 ± 0.4	0.018 *
Triglyceride (mg/dL) (mmol/L)	148.1 ± 110.6 1.7 ± 1.2	140.5 ± 97.4 1.6 ± 1.1	0.250
Estimated glomerular filtration rate (mL/s per 1.73 m^2^)	78.4 ± 20.4	78.8 ± 22.4	0.777
**Medication**			
Aspirin (*n*, %)	62 (11.6)	60 (13.0)	0.587
Antihypertensive drugs (*n*, %)	449 (84.2)	415 (90.0)	0.009 *
Metabolic syndrome (*n*, %)	200 (37.5)	198 (43.0)	0.093
**Outcome**			
10-year ASCVD (%) ^a^	13.8	20.6	<0.001 *

Abbreviations: ASCVD, atherosclerotic cardiovascular disease; ARR, aldosterone to renin ratio; EH, essential hypertension; PA, primary aldosteronism; PAC, plasma aldosterone concentration; PRA, plasma renin activity. ^a^ ASCVD (atherosclerotic cardiovascular disease): myocardial infarction, other coronary heart disease, or stroke [7].

**Table 2:** The validation of the ACC/AHA guidelines on 10-year ASCVD risk in middle-aged hypertensive patients with EH and PA.

	EH (*n* = 533)	PA (*n* = 461)	*p*
Recommend statin use by ACC/AHA guidelines	332 (62.3)	305 (66.2)	0.229
Predicted 10-year ASCVD risk ^a^ (%)			
ACC/AHA estimated 10-year ASCVD risk	10.9 ± 7.0	12.8 ± 8.3	<0.001 *
ACC/AHA estimated 10-year ASCVD risk > 7.5%	14.6 ± 6.2	16.5 ± 7.4	<0.001 *
**ASCVD Risk prediction** (AUROC, 95% CI)			
ACC/AHA estimated 10-year ASCVD risk	0.64 (0.58–0.71)	0.76 (0.70–0.82)	
ACC/AHA estimated 10-year ASCVD risk > 7.5%	0.61 (0.54–0.67)	0.65 (0.59–0.71)	
Recommend statin use by ACC/AHA guidelines	0.93 (0.91–0.95)	0.94 (0.91–0.96)	

Abbreviations: ACC, American College of Cardiology; AHA, American Heart Association; ASCVD, atherosclerotic cardiovascular disease; AUROC, area under receiver operating characteristic curve; EH, essential hypertension; PA, primary aldosteronism. ^a^ ASCVD (atherosclerotic cardiovascular disease): myocardial infarction, other coronary heart disease, or stroke [7].

## Supplementary Materials

The online-only Data Supplement is available at http://www.mdpi.com/2077-0383/7/11/382/s1. Figure S1: Major recommendations for statin therapy on 10-year ASCVD prevention under ACC-AHA guideline recommendations. Table S1. The sensitivity and specificity for 10-year ASCVD in middle-aged hypertensive patients with PA and EH by the recommendation of statin use based on ACC-AHA guideline. Table S2. Demographic comparisons of enrollees between PAC < 60 ng/dL and PAC ≥ 60 ng/dL.
