# Supplementary material for: Plasma Aldosterone Concentration as a Determinant for Statin Use among Middle-Aged Hypertensive Patients for Atherosclerotic Cardiovascular Disease
Source: J Clin Med. 2018 Oct 24;7(11):382. doi: 10.3390/jcm7110382 (PMC6262476; doi:10.3390/jcm7110382)
Supplement: Supplementary file 1 [file jcm-07-00382-s001.pdf]

Supporting Information

Supplement to: Plasma Aldosterone Concentration as a Determinant for Statin Use among Middle-Aged Hypertensive Patients on Atherosclerosis Cardiovascular Disease

Figure S1: Major recommendations for statin therapy on 10-year ASCVD prevention under the ACC-AHA guideline recommendations

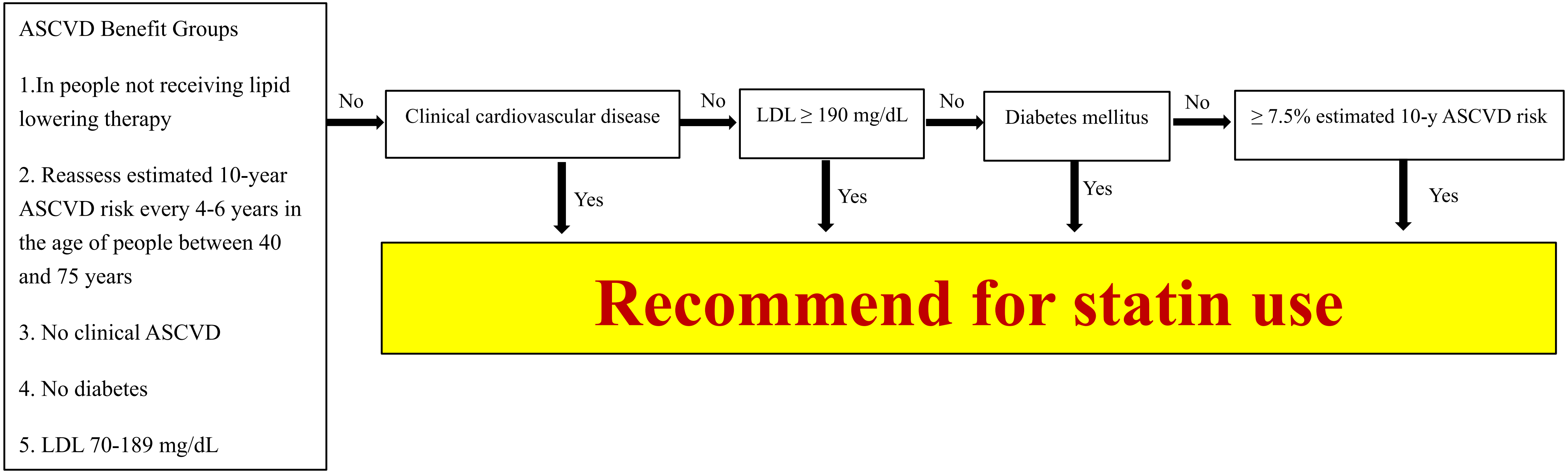

Abbreviations: ACC, American College of Cardiology; AHA, American Heart Association; ASCVD, atherosclerotic cardiovascular disease; LDL, low-density lipoprotein

Table S1. The sensitivity and specificity for 10-year ASCVD in middle-aged hypertensive patients with PA and EH by the recommendation of statin use based on the ACC/AHA guidelines

	**Discriminative cut-off value**	**Sensitivity (95% CI)**	**Specificity (95% CI)**	**AUROC (95% CI)**	***p***
**PA (*n* = 461)**					
**10-year ASCVD risk**	7.5%	98.0% (95.8, 99.3)	88.5% (82.4, 93.0)	0.94 (0.91, 0.96)	<0.001
**EH (*n* = 533)**					
**10-year ASCVD risk**	7.5 %	95.5% (92.7, 97.4)	91.0% (86.2, 94.6)	0.93 (0.91, 0.95)	<0.001

Abbreviations: ACC, American College of Cardiology; AHA, American Heart Association; ASCVD, atherosclerotic cardiovascular disease; AUROC, area under receiver operating characteristic curve; CI, confidence interval; EH, essential hypertension; PA, primary aldosteronism.

Table S2. Demographic comparisons of enrollees between the PAC < 60 ng/dL and PAC ≧ 60 ng/dL groups.

	**PAC < 60ng/dL (*n* = 848)**	**PAC ≧ 60ng/dL (*n* = 146)**	***p***
Age (years)	52.6 ± 9.4	50.2 ± 9.3	0.921
Male gender (%)	411 (48.5)	60 (41.1)	0.116
Body mass index (Kg/m^2)	25.5 ± 3.9	24.8 ± 3.5	0.168
Obesity (%)	101 (11.9)	12 (8.2)	0.247
Waist circumference (cm)	83.5 ± 10.8	80.8 ± 11.4	0.452
Systolic blood pressure (mmHg)	148.2 ± 22.1	150.5 ± 21.6	0.753
Diastolic blood pressure (mmHg)	88.2±13.7	89.8±14.4	0.427
Smoking status (%)	106 (12.5)	26 (17.8)	0.107
Diabetes mellitus (n,%)	106 (12.5)	23 (15.8)	0.344
Left ventricular hypertrophy (n,%)	112 (13.2)	31 (21.2)	0.015*
**Laboratory data**			
PAC (ng/dL)	31.2 ± 13.0	104.1 ± 144.1	<0.001*
(nmol/L)	0.9 ± 0.4	2.9 ± 4.0	
PRA (ng/mL/h)	2.6 ± 6.3	2.8 ± 8.3	<0.001*
(pmol/L/h)	61.6 ± 149.3	66.4 ± 196.7	
Log ARR	1.7 ± 0.9	2.3 ± 0.9	0.491
Potassium (mmol/L)	3.9 ± 0.7	3.5 ± 0.8	0.016*
(mEq/L)	3.9 ± 0.7	3.5 ± 0.8	
Fasting blood glucose (mg/dL)	99.9 ± 22.3	95.6 ± 18.2	0.007*
(mmol/L)	5.5 ± 1.2	5.3 ± 1.0	
Total cholesterol (mg/dL)	196.1 ± 37.0	195.1 ± 36.9	0.998
(mmol/L)	5.1 ± 1.0	5.1 ± 1.0	
Low density lipoprotein (mg/dL)	117.2 ± 32.4	115.8 ± 31.4	0.632
(mmol/L)	3.0 ± 0.8	3.0 ± 0.8	
Low density lipoprotein ≥ 190 mg/dL (n,%)	17 (2.0)	4 (2.7)	0.796
High density lipoprotein (mg/dL)	47.1 ± 13.1	47.5 ± 12.6	0.580
(mmol/L)	1.2 ± 0.3	1.2 ± 0.3	
Triglyceride (mg/dL)	147.2 ± 108.3	129.3 ± 79.1	<0.001*
(mmol/L)	1.7 ± 1.2	1.5 ± 0.9	
Estimated glomerular filtration rate (mL/s per 1.73 m^2)	80.9 ± 23.7	78.5 ± 31.1	<0.001*
**Metabolic syndrome (n,%)**	345 (40.7)	53 (36.3)	0.365
**Outcome**			
10-year ASCVD (%)[a]	11.8	11.4	0.601

Abbreviations: ASCVD, atherosclerotic cardiovascular disease; ARR, aldosterone to renin ratio; EH, essential hypertension; PA, primary aldosteronism; PAC, plasma aldosterone concentration; PRA, plasma renin activity.

[a] ASCVD (atherosclerotic cardiovascular disease): myocardial infarction, other coronary heart disease, or stroke.
